# Nudges to reason: not guilty

**DOI:** 10.1136/medethics-2017-104639

**Published:** 2017-11-18

**Authors:** Neil Levy

**Affiliations:** 1 Department of Philosophy, Macquarie University, Sydney, New South Wales, Australia; 2 Uehiro Centre for Practical Ethics, University of Oxford, Oxford, UK

**Keywords:** autonomy, behaviour modification

I am to grateful to Geoff Keeling for his perceptive response^1^ to my paper.^2^ In this brief reply, I will argue that he does not succeed in his goal of showing that nudges to reason do not respect autonomy. At most, he establishes only that such nudges *may* threaten autonomy when used in certain ways and in certain circumstances. As I will show, this is not a conclusion that should give us grounds for particular concerns about nudges.

Before turning to this issue, let me correct some small issues of interpretation of my paper. Keeling takes me to be committed to three descriptive claims: (1) that we have entered a post-truth era, (2) that our problem with the rational assessment of evidence is explained by or stems from the backfire effect and (3) that nudges to reason work by exploiting affective mechanisms. I am not committed to accepting any of these claims. I am not competent to assess whether we live in an age that is qualitatively different from previous eras, so far as our responsiveness to evidence is concerned. That is a question for historians and political scientists to settle. I am committed to claiming only that the label ‘post-truth’ identifies a genuine and serious problem, not necessarily a novel phenomenon. I deny that the backfire effect is at the heart of this genuine and serious problem. The backfire effect is not a mechanism: it is rather (at most) the upshot of mechanisms. There is extensive and undisputed evidence for a variety of phenomena (motivated reasoning, the confirmation bias, etc), which together explain resistance to good evidence. The backfire effect is illustrative of the problem not itself the problem. Similarly, my discussion of how affective mechanisms are partially constitutive (rather than independent) of reasoning is intended to be illustrative. It is not intended to suggest that all the non-deliberative or non-conscious ways in which we process information are affective.

I now turn to the heart of the matter. Keeling argues that nudges to reason may threaten autonomy. The explicit claim seems to be that they are deceptive, though there may be a second implicit claim: that even when they are not deceptive, they may nevertheless threaten autonomy. I completely agree with Keeling that nudges to reason may threaten autonomy even if they do not bypass reasoning mechanisms. There are other ways of threatening autonomy. However, that’s no special problem for defenders of nudges. After all, those who attack nudges aim to identify a problem with them that entails that they are somehow suspicious *compared with other ways of changing minds* (like rational argument). Nudges can be used deceptively; that’s a property they share with every other way of changing minds.

Keeling’s ‘newspaper’ argument seems to be intended to illustrate how nudges to reason may be used deceptively, but it does not actually seem to involve any deception. Perhaps the idea is that the government has a secret policy to require newspapers to publish articles from a different political perspective without informing readers either that they do this or which articles are concerned. If that’s the idea, then the objection collapses back into the first: advocates of nudges need not, and do not, hold that we should engage in deception. If the claim is instead that requiring newspapers non-deceptively to publish articles from different political perspectives is disrespectful of people’s autonomy, then I simply don’t accept it. It does not fail ‘to take seriously those readers as individuals capable of making informed decisions about which political evidence they give most weight to’, since it leaves it up to the reader to decide whether to consume the information. Coercion threatens autonomy, but making information available is not coercive. If the reader does not share my intuition here, no matter: it suffices to point out that any problem with exposure to unwanted information is not specific to nudges: it applies with equal force to argument.

Nudges to reason surely can be used in ways that threaten autonomy. That does not distinguish them from other ways of addressing ourselves to rational agents. It is disrespectful to deceive and to coerce, whatever the cognitive mechanisms we use in these ways.
